# Assessing COVID-related concerns and their impact on antenatal and delivery care among pregnant women living with HIV in Kenya: a brief report

**DOI:** 10.1186/s12884-023-06216-x

**Published:** 2024-01-09

**Authors:** Catherine Wexler, May Maloba, Sharon Mokua, Shadrack Babu, Nicodemus Maosa, Vincent Staggs, Kathy Goggin, Harshdeep Acharya, Emily A. Hurley, Sarah Finocchario-Kessler

**Affiliations:** 1 University of Kansas Medical Center, Kansas, USA; 2 Global Health Innovations, Nairobi, Kenya; 3 Kenya Medical Research Institute, Nairobi, Kenya; 4 Health Services and Outcomes Research, Children’s Mercy Kansas City, Kansas, USA; 5 University of Missouri - Kansas City Schools of Medicine and Pharmacy, Kansas, USA

**Keywords:** Prevention of mother to child transmission of HIV (PMTCT), HIV, Kenya, HITSystem, COVID-19

## Abstract

**Background:**

Some studies indicate that pregnant Kenyan women were concerned about Coronavirus disease 2019 (COVID-19) exposure during maternity care. We assessed concern regarding COVID-19 exposure and any impact on antenatal care (ANC) enrollment and/or hospital delivery among pregnant women living with human immunodeficiency virus (HIV) in Kenya.

**Methods:**

Data were collected from 1,478 pregnant women living with HIV enrolled in prevention of mother to child transmission of HIV (PMTCT) care at 12 Kenyan hospitals from October 2020 to July 2022. Surveys were conducted when women first presented for PMTCT services at the study hospital and asked demographic questions as well as items related to concerns about COVID-19. A 5-point Likert scale (strongly disagree to strongly agree) assessed concerns about COVID-19 exposure and travel challenges. Gestational age at PMTCT enrollment, number of ANC appointments attended, and delivery location were compared among women who expressed COVID-19 concerns and those who did not.

**Results:**

Few women reported delaying antenatal care (4.7%), attending fewer antenatal care appointments (5.0%), or having concerns about a hospital-based delivery (7.7%) because of COVID-19. More (25.8%) reported travel challenges because of COVID-19. There were no significant differences in gestational age at enrollment, number of ANC appointments, or rates of hospital-based delivery between women with concerns about COVID-19 and those without,

**Conclusion:**

Few pregnant women living with HIV expressed concerns about COVID-19 exposure in the context of routine ANC or delivery care. Women with and without concerns had similar care seeking behaviors. The recognized importance of routine ANC care and facility-based deliveries may have contributed to these positive pregnancy indicators, even among women who worried about COVID-19 exposure.

**Trial registration:**

www.clinicaltrials.gov identifier NCT04571684.

## Introduction

For pregnant women living with human immunodeficiency virus (HIV), prevention of mother to child transmission of HIV (PMTCT) services ideally start at the confirmation of pregnancy and include provision of antiretroviral treatment (ART) and routine viral load monitoring to ensure treatment efficacy, a recommended 8 antenatal care (ANC) visits, management of labor and delivery, provision of infant postnatal ARV prophylaxis, and early infant diagnosis of HIV (EID) through routine infant HIV testing through 18 months [1].

Coronavirus disease 2019 (COVID-19) has posed unprecedented challenges to HIV care globally and in Kenya. The first case of COVID-19 was recorded in Kenya in March of 2020 [2]. In March of 2020, the Kenyan Ministry of Health released guidelines on HIV service provision in the context of COVID-19 including immediate isolation of anyone presenting with a cough, reduced clinic hours, staggered appointment times throughout the day, and reduction of clinic attendance and drug pickup frequency [3]. These measures, combined with lockdowns and curfews, COVID-related reductions in income, lack of personal protective equipment, and increased difficulty obtaining public transport due to restricted passenger numbers were expected to contribute to increased challenges accessing health facilities and suboptimal clinical outcomes.

Data on clinical care utilization in Kenya suggest varying impacts of COVID-19. Some studies have noted reductions in hospital births (11%), admissions of children under (59%) and over (25–29%) five years old from May of 2020 to March of 2021 [4]; declines in HIV testing, ART initiation (by 50%), and medication and appointment adherence from March to June of 2020 [5]; and higher odds of delayed ANC during the early phases of the pandemic (July – November 2020) compared to pre-pandemic. Other studies, however, have found no difference in the number of ANC appointments attended and no difference in hospital births, family planning attendance, post-abortion care and pentavalent-1 immunization between pre-pandemic and early pandemic (March-June 2020) [6–8].

We assessed the level of COVID-19 concern and its impact on PMTCT service utilization among pregnant women living with HIV at 12 government health facilities in Kenya.

## Methods

### Study setting and eligibility

This study was nested within a larger randomized control trial evaluating the impact of the HITSystem 2.1 at 12 hospitals (6 intervention, 6 control) in Kenya (NCT04571684) [9]. The HITSystem is an eHealth system that uses electronic alerts for providers and SMS for patients to support retention in PMTCT and EID services including PMTCT appointment attendance, hospital-based delivery, and early infant testing [9–11]. Study hospitals were in Siaya (n = 8), Mombasa (n = 2), and Kilifi (n = 2) counties. Pregnant women living with HIV were eligible for this analysis if they presented for ANC care by 36 weeks gestation at one of the study hospitals between October 1, 2020 and July 5, 2022.

Women completed a baseline survey to assess sociodemographic factors, preferences and experiences with male partner support, motivation, and self-efficacy for accessing PMTCT services, and COVID-19 concerns. COVID-19 concerns/challenges [“I delayed coming for ANC care because of concerns related to COVID-19 exposure” (delayed ANC); “I do not want to attend the clinic as frequently for antenatal care because I am worried about COVID-19 exposure” (less frequent ANC); “I am worried that delivering in a hospital will expose me and my child to COVID-19” (Hospital based delivery); and “Travel to and from the hospital has become more challenging (cost and/or availability of transportation) as a result of COVID-19” (Travel challenges)] were assessed on a 5-point Likert scale (strongly agree to strongly disagree).

Clinical data were prospectively tracked at each clinical visit (ANC visit of ART refill appointment) and documented in the HITSystem (intervention sites) or a parallel electronic database – without the algorithm driven alerts (control sites). Gestational age at PMTCT enrollment was documented at study enrollment. Each ANC/PMTCT appointment was documented within the HITSystem/Control system at the time of the appointment. Delivery status and location were documented at the time of delivery or at the first appointment after delivery. Study staff followed up with participant within one month of their estimated delivery date if no delivery information was documented. Data were exported from the HITSystem or ControlSystem in Excel format for data analysis.

### Outcomes

The primary outcomes were gestational age at PMTCT enrollment, number of ANC appointments attended from the time of PMTCT enrollment to delivery, and delivery location (health facility vs. other) among women who expressed concerns with COVID-19 vs. those without concerns. Secondary outcomes included sociodemographic associations with COVID-19 concerns and changes in COVID-19 concern over time.

### Analyses

Responses to items assessing COVID-19 concerns/challenges were aggregated into agree (strongly agree, agree) and do not agree (neutral, disagree, strongly disagree). Descriptive statistics were used to describe participants’ sociodemographic characteristics, clinical characteristics and rates of COVID-19 concern. To assess changes in COVID-19 concern over time, dates of enrollment were broken into quarters, with quarter 1 starting October 1, 2020, through December 31, 2020 and each subsequent quarter representing a 3-month period. Percentage expressing concern were calculated among enrollees in each quarter. Chi-square or Fisher’s Exact tests (only in cases where observed values were < 5) were conducted to assess associations between demographic variables and levels of COVID-19 concern/transportation challenges and to assess differences in rates of gestational age at enrollment and hospital-based delivery among women who expressed concerns/transportation challenges, compared with those who did not. Chi-Square tests were used to compare the percentage of women who attended the guideline-recommended minimum of at least 4 ANC visits and those who did not among: (1) women who expressed concern about ANC appointment attendance and those who did not and (2) women who expressed travel challenges and those who did not. This analysis was limited to women who had delivered to minimize the impact of recent enrollments on number of prenatal appointments (e.g., a woman enrolled in the past month at 20 weeks gestation would be expected to have fewer ANC appointments documented).

## Results

A total of 1,478 women were enrolled in the study at the time of data analysis. At study enrollment, mean participant age was 30.0 years (standard deviation (SD): 5.9), mean gestational age was 18 weeks + 3 days (SD: 7.8), and mean time on ART was 4.1 years (SD: 3.7). Table 1 shows demographic characteristics of participants enrolled at baseline.

**Table 1:** Participant Demographic Variables and Level of COVID-19 Concern

		I delayed coming for ANC care because of concerns related to COVID-19 exposure			I am worried that delivering in a hospital will expose me and my child to COVID-19			Travel to and from the hospital has become more challenging as a result of COVID-19			I do not want to attend the clinic as frequently for antenatal care because I am worried about COVID-19 exposure		
	Total	Agree	Do not agree		Agree	Do not agree		Agree	Do not agree		Agree	Do not Agree	
	*N* (%)	*N* (%)	*N* (%)	*P*	*N*	*N*	*P*	*N*	*N*	*P*	*N*	*N*	*P*
**Age**				*P* = 0.77			*P* = 0.38			*P* = 0.55			*P* = 0.03
24	257 (17.6%)	11 (4.3%)	243 (95.7%)		23 (9.0%)	232 (91.0%)		70 (27.2%)	187 (72.8%)		20 (7.8%)	236 (92.2%)	
24	1205 (82.4%)	57 (4.7%)	1145 (95.3%)		89 (7.4%)	1113 (92.6%)		305 (25.5%)	893 (74.5%)		53 (4.5%)	1138 (95.5%)	
Education				*p* = 0.87a			*p* = 0.78			*p* = 0.08			*p* = 0.95a
No school	57 (3.9%)	4 (7.1%)	52 (92.9%)		5 (8.8%)	52 (91.2%)		19 (33.3%)	38 (66.7%)		2 (3.6%)	53 (96.4%)	
Partial primary	454 (30.8%)	20 (4.4%)	432 (95.6%)		39 (8.6%)	414 (91.4%)		124 (27.6%)	326 (72.4%)		20 (4.4%)	431 (95.6%)	
Completed primary	480 (32.6%)	24 (5.0%)	455 (95.0%)		31 (6.5%)	447 (93.5%)		102 (21.3%)	377 (78.7%)		25 (5.2%)	452 (94.8%)	
Partial secondary	188(12.8%)	8 (4.3%)	180 (95.7%)		14 (7.4%)	174 (92.6%)		53 (28.2%)	135 (71.8%)		10 (5.4%)	175 (94.6%)	
Completed secondary or beyond	196 (13.3%)	13 (5.1%)	279 (94.9%)		24 (9.3%)	268 (90.7%)		80 (29.9%)	212 (70.1%)		16 (5.5%)	274 (94.5%)	
HIV Disclosure Status				*p* = 0.66			*p* = 0.82			*p* = 0.84			*p* = 0.91
Anyone	1286 (87.2%)	59 (4.6%)	1221 (95.4%)		98 (7.6%)	1186 (92.4%)		329 (25.7%)	952 (74.3%)		64 (5.0%)	1209 (95.0%)	
No one	188 (12.8%)	10 (5.3%)	178 (94.7%)		15 (8.1%)	170 (91.9%)		49 (26.3%)	137 (73.7%)		9 (4.8%)	177 (95.2%)	
Relationship Status				*p* = 0.48a			*p* = 0.70a			*p* = 0.90			p-0.92a
Single	100 (6.8%)	7 (7.0%)	93 (93.0%)		8 (8.1%)	91 (91.9%)		28 (28.0%)	72 (72.0%)		5 (5.0%)	95 (95.0%)	
Married	1211 (82.0%)	54 (4.5%)	1151 (95.5%)		91 (7.5%)	1118 (92.5%)		306 (25.4%)	900 (74.6%)		59 (4.9%)	1138 (95.1%)	
Unmarried, but in a relationship	62 (4.2%)	2 (3.2%)	60 (96.8%)		3 (4.9%)	58 (95.1%)		17 (27.4%)	45 (72.6%)		3 (4.9%)	58 (95.1%)	
Separated or divorced	62 (3.7%)	4 (6.5%)	58 (93.5%)		6 (9.8%)	55 (90.2%)		15 (25.0%)	45 (75.0%)		3 (4.8%)	59 95.2%)	
Widowed	42 (2.8%)	2 (4.8%)	40 (95.2%)		5 (11.9%)	37 (88.1%)		13 (31.0%)	29 (69.0%)		3 (7.1%)	39 (92.9%)	
Partner's HIV status?				*p* = 0.61			*p* = 0.19			*p* = 0.034			*p* = 0.48
HIV +	655 (44.5%)	30 (4.6%)	623 (95.4%)		49 (7.5%)	606 (92.5%)		162 (24.8%)	492 (75.2%)		31 (4.8%)	616)95.2%)	
HIV-	372 (25.3%)	14 (3.8%)	356 (96.2%)		28 (7.5%)	343 (92.5%)		107 (28.9%)	263 (71.1%)		23 (6.2%)	346 (93.8%)	
Unknown	325 (22.1%)	16 (5.0%)	307 (95.0%)		21 (6.5%)	301 (93.5%)		69 (21.4%)	254 (78.6%)		12 (3.7%)	309 (96.3%)	
No current partner	120 (8.2%)	8 (6.7%)	112 (93.3%)		15 (12.6%)	104 (87.4%)		40 (33.9%)	78 (66.1%)		7 (5.8%)	113 (94.2%)	
Average weekly household income				*p* = 0.16			*p* = 0.13			*p* = 0.16			*p* = 0.29
Less than 500 KES	300 (20.4%)	22 (7.3%)	278 (92.7%)		14 (4.7%)	284 (95.3%)		62 (20.9%)	235 (79.1%)		15 (5.1%)	282 (94.9%)	
500–750 KES	323 (21.9%)	14 (4.3%)	308 (95.7%)		26 (8.1%)	296 (91.9%)		84 (26.0%)	239 (74.0%)		10 (3.1%)	313 (96.9%)	
750–1,000 KES	318 (21.6%)	13 (4.1%)	302 (95.9%)		30 (9.5%)	287 (90.5%)		92 (29.1%)	224 (70.9%)		21 (6.7%)	291 (93.3%)	
1,000–2,500 KES	319 (21.7%)	11 (3.5%)	306 (95.5%)		29 (9.1%)	289 (90.9%)		88 (27.6%)	231 (72.4%)		18 (5.7%)	298 (94.3%)	
2,500 KES	212 (14.4%)	8 (3.8%)	204 (96.2%)		13 (6.1%)	199 (93.9%)		50 (23.8%)	160 (76.2%)		9 (4.3%)	200 (95.7%)	
Previous experience with PMTCT/EID													*p* = 0.21
Yes	831	43 (5.2%)	786 (94.8%)	*P* = 0.30	62 (7.5%)	769 (92.5%)	*P* = 0.73	216 (26.1%)	612 (73.9%)	*P* = 0.75	36 (4.4%)	789 (95.6%)	
No	647	26 (4.0%)	617 (96.0%)		51 (7.9%)	591 (92.1%)		163 (25.5%)	480 (74.5%)		37 (5.8%)	601 (94.2%)	

**Fig. 1:**
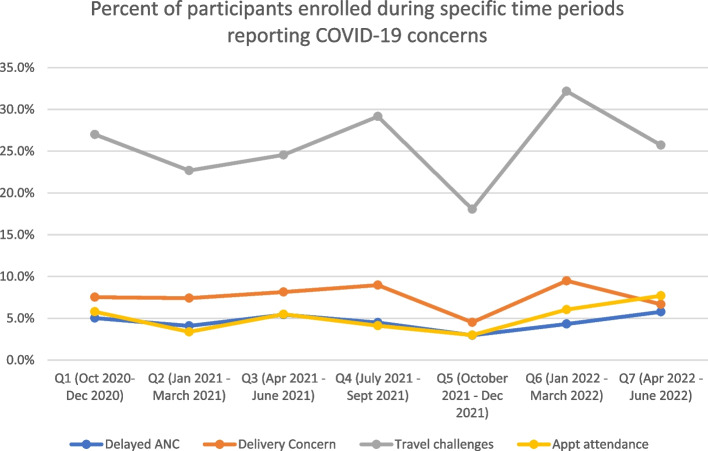
COVID-19 concern at enrollment over time

A total of 69 (4.7%) of women agreed with the statement regarding delayed ANC care, and this ranged from a low of 3.0% in Q3 to high of 5.8% in Q7. A total of 73 (5.0%) women agreed with the statement regarding less frequent ANC care, ranging from 3.0% in Q5 to 7.7% in Q7. A total of 113 (7.7%) women agreed with concerns about a hospital-based delivery, ranging from 4.5% in Q5 to 9.5% in Q6, and 379 (25.8%) agreed with the statement about increased travel challenges, ranging from a low of 18% in Q5 to 29.1% in Q1, see Fig. 1 for COVID-19 concern over time.

Of the demographic variables assessed, only partner HIV status was associated with travel challenges to the hospital and mother age > 24 was associated with increased likelihood of concern regarding frequency of ANC appointments. No other demographic variables were associated with any measure of COVID-19 concern, Table 1.

### Gestational age at enrollment

Of the 1,478 women enrolled at baseline, there was no difference in gestational age at enrollment between women who agreed with the statement about delaying ANC care due to concerns about COVID-19 exposure vs those who did not agree. There was no difference in gestational age at enrollment among women who agreed with the statement regarding increased transportation challenges due to COVID-19 vs those who did not.

A total of 1,127 women (76.3%) had delivered their child at the time of data analysis and are included in delivery location and appointment attendance measurements.

### Delivery location

A total 1,123 (76%) women had a location of delivery documented, of whom 1,062 (94.6%) delivered in a health facility. There was no difference in delivery location among women who expressed COVID-19 concerns related to hospital delivery: (82/84, 97.6%) vs those who did not (976/1,034, 94.4%, Fisher exact test p = 0.311). Furthermore, there was no difference between rates of facility delivery among women who indicated travel challenges (267/287, 93.0%) and those who did not (789/829, 95.2%; Fisher exact test p = 0.173).

### Appointment attendance

Among women who had delivered, 578 (55.6%) attended at least 4 ANC appointments. Among women who had delivered and agreed with the statement regarding less frequent ANC care due to COVID-19 concerns*,* 48.1% attended at least 4 ANC visits compared to 55.1% among women who did not agree with the statement, p = 0.32. Among women who had delivered and agreed with the statement regarding travel challenges, 50.9% attended at least 4 ANC appointments compared to 55.9% of women who did not agree with the statement regarding travel challenges, p = 0.14.

## Discussion

While some studies have found impacts of COVID-19 on HIV care seeking [4, 5], others have found minimal or transient effects of COVID-19 on service delivery in Kenya [6, 7]. Our results indicate that by October of 2020, there were low rates of COVID-19 concern. More women expressed travel challenges, likely reflecting mandates reducing the number of passengers on public buses, increased fares, and travel curfews [12]. However, there were minimal differences in PMTCT service provision among women with and without concerns and travel challenges.

By this point in the pandemic, many of the early and stringent restrictions related to COVID-19 were relaxed and the health system had developed strategies to mitigate the impact of restrictions on continuity of healthcare services [8]. Thus, it is possible that levels of concern and its impact on service uptake by October of 2020 was already reduced, and evaluating similar metrics at an earlier timepoint would show increased concern. While we do not have direct data to compare clinical indicators assessed in this study to the pre-pandemic period, our data compared with pre-pandemic studies suggests that changes in PMTCT care seeking was minimal. While the mean of 3.8 ANC appointments per women observed in this study is below national targets, it is similar to reported numbers of ANC visits achieved in pre-pandemic periods (range: 3.3–4.1 appointments) [11]. Similarly, the gestational age at enrollment reported in our study (18.5 weeks) and rate of facility-based delivery (94.6%) are similar to pre-pandemic rates (18.1–22.3 weeks; 63%-96%) [11]. Furthermore, previous analyses comparing ANC appointment attendance and hospital based deliveries among women living with and without HIV from January 2019 to November of 2020 showed no significant differences between pre- and post- pandemic timepoints [8].

## Limitations

The data collection period and lack of direct pre-pandemic data are limitations to the study.

## Conclusion

Our data suggests that by October of 2020, the COVID-19 pandemic had minimal impact on PMTCT care seeking behavior among pregnant women living with HIV. The recognized importance of routine ANC care and facility-based deliveries may have contributed to these positive pregnancy indicators, even amongst women who worried about COVID-19 exposure.

## Data Availability

The datasets used and/or analysed during the current study available from the corresponding author on reasonable request.
